# Non-communicable diseases, psychosocial wellbeing, and quality of life in Ga Mashie, Accra, Ghana: analysis from a community-based cross-sectional study

**DOI:** 10.1186/s12889-025-22227-z

**Published:** 2025-03-19

**Authors:** Kafui Adjaye-Gbewonyo, Irene Akwo Kretchy, Leonard Baatiema, Carlos S. Grijalva-Eternod, Olutobi Adekunle Sanuade, Samuel Amon, Hassan Haghparast-Bidgoli, Raphael Baffour Awuah, Swaib Abubaker Lule, Sedzro Kojo Mensah, Sandra Boatemaa Kushitor, Mawuli Komla Kushitor, Daniel Kojo Arhinful, Edward Fottrell

**Affiliations:** 1 Institute for Lifecourse Development, University of Greenwich, London, UK; 2 Department of Pharmacy Practice and Clinical Pharmacy, School of Pharmacy, University of Ghana, Accra, Ghana; 3 Department of Health Policy, Planning and Management, School of Public Health, University of Ghana, Accra, Ghana; 4 Centre for Tropical Medicine and Global Health Research, Nuffield Department of Medicine, University of Oxford, Oxford, UK; 5 Institute for Global Health, University College London, 30 Guilford Street, London, WC1N 1EH UK; 6 London School of Hygiene & Tropical Medicine, London, UK; 7 Department of Population Health Sciences, Division of Health System Innovation and Research, Spencer Fox Eccles School of Medicine, University of Utah, Salt Lake City, United States of America; 8 Noguchi Memorial Institute for Medical Research, University of Ghana, Accra, Ghana; 9 Vital Strategies, New York, United States of America; 10 Regional Institute for Population Studies, University of Ghana, Accra, Ghana; 11 Department of Community Health, Ensign Global College, Kpong, Ghana; 12 Department of Food Science and Centre for Sustainability Studies, Stellenbosch University, Stellenbosch, South Africa; 13 Department of Health Policy, Planning and Management (HPPM), Fred N. Binka School of Public Health, University of Health and Allied Sciences (UHAS), Ho, Ghana

**Keywords:** Non-communicable diseases, Quality of life, Psychosocial wellbeing, Diabetes, Hypertension, Obesity

## Abstract

**Background:**

The burden of non-communicable diseases (NCDs), such as diabetes, hypertension, and obesity, is increasing globally, particularly in low- and middle-income countries. This has implications for physical, psychological and social aspects of health and wellbeing among individuals living with NCDs. This study sought to examine relationships between NCDs, quality of life (QoL) and psychosocial wellbeing in the urban community of Ga Mashie, Accra, Ghana.

**Methods:**

A representative household survey was conducted among adults aged 25 years and over in Ga Mashie. Data were collected on self-reported NCD diagnoses and objectively measured random blood glucose, blood pressure and anthropometrics; sociodemographic characteristics; and health-related QoL and psychosocial wellbeing. Relationships between QoL, psychosocial wellbeing and diabetes, hypertension and obesity were examined using bivariate analyses and multivariable regressions comparing outcomes to those who did not have any of these conditions or any other self-reported NCD diagnosis.

**Results:**

Data were gathered from 854 adults. Individuals with diabetes, hypertension and obesity had significantly reduced measures of QoL outcomes compared to those without these conditions or any other reported NCD. In particular, they were significantly less likely to report being satisfied or very satisfied with their health [Risk Ratios: 0.79 (95% CI: 0.63–0.98), 0.87 (95% CI: 0.79–0.95) and 0.87 (95% CI: 0.77–0.97) for diabetes, hypertension, and obesity, respectively]. They also had lower scores in the physical health domain of QoL compared to those with no known NCD [diabetes β -8.27 (95% CI: -13.55– -2.99); hypertension − 2.32 (95% CI: -4.84–0.21) and obesity − 2.74 (95% CI: -5.15– -0.32)]. Compared to the healthy comparison group, differences were more pronounced among those with a prior diagnosis of diabetes or hypertension than among those identified with raised blood glucose or raised blood pressure in our survey, but no prior diagnosis. Differences in indicators of psychosocial wellbeing such as social support, and psychological distress were not observed.

**Conclusion:**

Diabetes, hypertension and obesity were associated with reduced QoL in Ga Mashie, Ghana. Further studies are needed to understand factors that influence health-related QoL among adults with NCDs, how these change over time, and to identify potential coping mechanisms that may influence this association.

**Supplementary Information:**

The online version contains supplementary material available at 10.1186/s12889-025-22227-z.

## Background

Chronic non-communicable diseases (NCDs) are the major causes of death, morbidity, loss of independence and diminished quality of life globally. It is estimated that NCDs accounted for 74% of global deaths in 2019 [1], and a high proportion of this number are reported from sub-Saharan Africa and other resource-poor regions of the world [2]. For example, studies have shown that the burden of NCDs, such as diabetes and hypertension, is increasing in low-middle income countries (LMICs), and yet, in these settings, there is less prioritization for NCD prevention and control, and access to screening and treatment services remains sub-optimal [3]. Many factors account for this rising burden, and chief among these factors are the demographic and nutrition transitions where populations in these settings are growing and simultaneously experiencing a rise in life expectancy (aging), westernization of diets and increase in sedentary lifestyles. However, policy and research attention seem skewed towards the epidemiological burden, although the rise in NCDs in these contexts also has far-reaching ramifications on the mental, economic, psychosocial, and overall wellbeing of the populace. The World Health Organisation (WHO) defines health as ‘a state of complete physical, social and mental wellbeing and not merely the absence of disease and infirmity’ [4]. Therefore, it is important to advance and extend understandings beyond the epidemiological burden to how the rise in NCDs impacts different aspects of health and wellbeing—the physical, social, and psychological.

Psychosocial factors such as stress, social support and psychological distress have been linked to several health outcomes. For example, social support has been associated with mental health, health behaviours and health service utilisation in Ghana and elsewhere [5–10]. Studies have also found associations between stress and mental ill health, unhealthy coping behaviours, as well as impaired wound healing, cardiovascular health and immune function [7, 11–16]. Likewise, psychological distress has been associated with reduced medication adherence [17, 18], while psychosocial wellbeing, which includes both emotional and social wellbeing [19], has been found to predict physical and mental health-related quality of life (QoL) among individuals with chronic conditions or impairment [20]. Furthermore, studies have reported that older people living with NCDs have significant reductions in QoL and independence [21]. The presence of more than one NCD is associated with worse QoL scores and greater difficulties in activities of daily living [22, 23]. Poor social support, income and educational levels have also been reported to be directly associated with mental health components of QoL [24–26]. Despite the increasing research and policy interest in measuring and studying QoL, the evidence-base on QoL and other psychosocial dimensions of health outcomes is still developing and this knowledge gap is wider in LMICs such as Ghana. To date, much research on psychosocial wellbeing and QoL has been conducted in high-income countries or outside the Africa region [27]. This situation raises critical questions beckoning further interrogation to understand the relationship between NCD conditions, QoL and psychosocial wellbeing in the African context. To address this question, this study explores the associations between diabetes, other NCDs (i.e. hypertension, obesity) and QoL and other psychosocial characteristics (perceived stress, psychological distress, social support) among adults aged 25 years and above in Ga Mashie, Accra, Ghana.

## Methods

### Study design

This analysis uses microdata from the household survey of the Contextual Awareness, Response and Evaluation: Diabetes in Ghana (CARE Diabetes) project [28] in order to examine risk for reduced psychosocial wellbeing and QoL in Ga Mashie according to NCD status. The survey was a community-based and representative household cross-sectional epidemiological study conducted in Ga Mashie in the Greater Accra Region of Ghana.

### Study setting and participants

Nine hundred and fifty-nine households were selected in Ga Mashie, a densely populated urban poor setting comprising two indigenous communities, namely James Town and Ussher Town. Ga Mashie is located on the southwestern coast of the Greater Accra Region and houses some of the oldest settlements in Accra. Trading, fishing and other fishing-related activities are the primary source of livelihood for members of the community [28]. Previous research has documented the presence of NCDs and their risk factors in Ga Mashie, including food and dietary risks [29], issues related to ageing [30], as well as cardiovascular risk factors and conditions such as stroke [31, 32].

A target sample size of 1,242 individuals was calculated based on the assumed diabetes prevalence of 5.0% [33], a precision of 2.0% and a design effect of 2.5. We assumed that each household in the study area would, on average, include two eligible adults, that around 40% of listed households would be empty or non-traceable households, and that there would be a 10% non-response rate. To ensure broad geographical representation of the 80 census enumeration areas (EAs) in Ga Mashie we used a simple random sampling technique to randomly select 12 households in each EA using the Ghana Statistical Service’s latest census in Ga Mashie [34], resulting in a final target of 959 households.

Eligibility criteria for this study included male and female adults aged ≥ 25 years who were permanent residents of the selected households, i.e., residing in the household for the past 12 months. The study excluded pregnant women or those who had given birth within the past six months as well as individuals who were unable to provide informed consent or had difficulty completing the survey, including those who were mentally incapacitated.

### Data collection procedure

Forty enumerators were recruited and trained on the survey tools and data collection procedures. This included processes and procedures in obtaining informed consent, conducting participant interviews, maintaining confidentiality, measuring and recording anthropometric, blood glucose, and blood pressure data, and using Open Data Kit (ODK) questionnaires on mobile devices. The enumerators were also trained on all detailed standard operating procedures to be followed by survey researchers during the field work.

During the survey, the team used electronic questionnaires on Android mobile devices to capture data at both household and individual levels. The devices were encrypted and password protected for security. Unique household identifiers for each household on the sampling list were assigned, along with recording of other identifying information such as structure and house numbers, and addresses. The household head reviewed the captured data and confirmed its accuracy. Separate questionnaires were used on the ODK for household and individual level data. All information was securely uploaded onto an organizational network analysis server for storage, cleaning, coding, and anonymization.

A pre-test of the survey tools and procedures was conducted among 50 households in the nearby La Dade-Kotopon Municipal area of Accra. Pre-testing largely confirmed appropriateness of tools and methods and only minor modifications were made to improve efficiency and data flow. Data from the pre-test were not included in the final survey data analysis.

### Measures

The primary outcomes for this analysis are QoL, perceived stress, psychological distress, and social support. The predictors of interest are diabetes status, hypertension, and obesity. Covariates include demographic indicators (e.g., age, sex, marital status, employment status) and socioeconomic indicators (e.g., education level, household wealth).

Details of the measures and analysis are provided below. Further details on the survey are described by Lule et al. [28], and materials are available from the corresponding author upon request.

#### Outcomes

##### Quality of life

Quality of life (QoL) was measured using the WHOQOL-BREF scale [35]. It is an abbreviated version of the WHOQOL-100, contains 26 items on a Likert scale and has been previously applied in Ghana [36]. The items measure four domains of QoL (physical, psychological, social, and environmental) and also include two standalone questions measuring overall perceptions of QoL and satisfaction with health. Scores for each of the domains of the WHOQOL-BREF were calculated following the methods outlined for computing domain scores in the WHOQOL User Manual [35]. These were transformed to a scale ranging from 0 to 100 with higher scores indicating better QoL. For the two standalone questions on overall perceptions of QoL and satisfaction with health (Items 1 and 2), the proportion of respondents selecting each response category was also used as a categorical measure.

##### Psychosocial wellbeing

Indicators of psychological and social wellbeing included psychological distress, social support and perceived stress as described below:

#### Psychological distress

Psychological distress was measured using the Psychological Distress Scale (PDS) developed and used in the Urban Health and Poverty Survey (EDULINK Wave III) conducted in Accra, Ghana in 2013. The scale captures questions on symptoms of anxiety and depression, and details of the nine items in the scale are described by Kushitor and colleagues [10]. An overall score ranging from 9 (low levels of distress) to 45 (high levels of distress) was calculated by reverse-coding the four positively worded items and then summing the responses on all nine items.

#### Perceived stress

Perceived stress was measured using the 10-item version of the Perceived Stress Scale (PSS-10) [12], which measures the frequency of experiencing feelings of stress over the past month. Items are on a five-point scale ranging from 0 (never) to 4 (very often). The four positively worded questions were reverse coded such that all questions represented increasing feelings of stress from 0 to 4. The responses were summed across questions and then the summed scores were averaged by dividing by 10 to represent average levels of stress from 0 to 4. Averages were taken to allow for potential comparisons across sub-domains of the scale.

Some studies have suggested a two-factor structure or two-dimensional model for the PSS-10 [37, 38], consisting of distress/lack of control (items 1, 2, 3, 6, 9 and 10) and lack of self-efficacy (items 4, 5, 7 and 8). While the main analyses used the full PSS-10 score, sensitivity analyses looking at these two dimensions separately were also conducted. Scores for each dimension were likewise summed and then averaged by the number of items to allow for comparisons.

#### Social support

Social support was measured using the three-item Oslo Social Support Scale (OSSS-3) [39]. Responses across the three items were summed to produce scores ranging from 3 to 14, with higher values indicating higher levels of social support. OSSS-3 scores were categorised following the method used by Kocalevent and colleagues [40] and Bøen and colleagues [41], with scores of 3 to 8 representing poor social support, scores of 9 to 11 representing moderate social support and scores of 12 to 14 representing strong social support.

#### Predictors

##### Non-communicable diseases

Data on NCDs was derived from direct measurement and from medical history based on the WHO STEPwise tool [42]. Diabetes included both those self-reporting a prior diagnosis of diabetes and those who had a random blood glucose measurement above 11.1 mmol/L at the time of the survey based on finger-prick capillary blood sample analysed with a point-of-care glucometer. While random blood glucose is not a diagnostic test for diabetes, it helps to estimate and assess diabetes risk in large epidemiological studies when other measures such as HbA1c and fasting blood glucose are difficult to obtain [43].

Hypertension included those either self-reporting a diagnosis of hypertension or those whose mean systolic blood pressure measured above 140 mmHg on the second and third reading or whose mean diastolic blood pressure measured above 90 mmHg in the second and third reading using a digital blood pressure monitor.

Obesity was defined as having a body mass index (BMI) equal to or above 30 kg/m^2^ as measured using standard protocols for height and weight measurement during the survey.

A healthy sample was defined for the purposes of this analysis as those who did not have any of the following: high blood pressure measurements, raised blood glucose measurements, BMI > 30 kg/m^2^, or reported any NCD diagnosis [diabetes, heart disease (angina or abnormal heart rhythm), stroke, chronic lung disease, hypertension (high blood pressure), cancer or malignant tumour, asthma, arthritis, kidney disease, liver disease, high blood cholesterol or obesity]. This resulted in a little over one-third of the sample (306 individuals) considered ‘healthy’. This sample was used as the comparison group.

#### Covariates

Sociodemographic characteristics were adapted from the Ghana Demographic and Health Survey [44]. For multivariable analyses, covariates included variables that may be associated with QoL in the community. Sex was dichotomised as male or female and age in years was included as a continuous variable. Education was condensed into three categories: no education, basic education (pre-school, primary, middle/junior high school) and secondary/senior high school/higher education. Marital status was grouped into three categories: currently married/living together, divorced/separated/widowed, and never married. Employment status was defined as either currently working or not currently working. Local status was a binary variable (always lived in the community versus migrant).

In terms of ethnicity, the majority of participants belonged to either the Ga-Dangme ethnic group or Akan ethnic groups; therefore, all other ethnicities were collapsed into an ‘Others’ category to avoid small numbers. Religion was grouped into four categories: no religion, Christianity, Islam, Traditional/spiritual/other.

A wealth index was derived from household assets using principal components analysis. Due to the sample size and distribution of wealth, we categorized households into tertiles of wealth and labelled them ‘most poor’, ‘poor’, and ‘least poor’ to reflect the relatively deprived nature of the community overall.

#### Analysis

Analyses were conducted in Stata 17 and all analyses used the survey commands to account for the sampling design and generate population estimates and 95% confidence intervals.

Descriptive statistics (e.g., means/proportions and 95% confidence intervals) were used to summarise the main outcomes, predictors, and covariates in the population. Bivariate analyses examined associations between the outcomes of interest and predictors. In bivariate analyses, Pearson’s chi-square was used to compare proportions while T-tests were used to compare means using simple linear regression.

Bivariate analyses with QoL indicators were used to determine which covariates to include in the models as control variables. Migrant/local status was not included in the models as a control variable as no significant differences were observed by that variable in any of the QoL measures.

Multivariable regression analyses explored the independent associations between the predictors and outcomes of interest while controlling for other covariates. Multivariable regressions were only run for the QoL outcomes as no significant differences were observed in indicators of psychosocial wellbeing by NCD status. For the categorical indicators on overall perceptions of QoL and satisfaction with health, these were dichotomised based on the distribution (e.g. good/very good versus remaining categories and satisfied/very satisfied compared to other categories). Poisson regression was used to estimate risk ratios for having good/very good perception of QoL and being satisfied/very satisfied with health. For the four domain scores of QoL (physical, psychological, social, and environmental), linear regression models were used.

## Results

From the original sample of 959 eligible households randomly selected from 80 EAs, 31% were not found and 1.5% refused to participate. Within the remaining 644 households there was a total of 1,007 eligible individuals, of whom 854 individuals in 629 households across 79 EAs participated in the survey (household response rate of 66%; individual response rate of 69%). Non-responders were more likely to be male, younger, and in the higher wealth tertile.

Table 1 presents descriptive statistics for the study population. Nearly two-thirds of the study population was female, and the mean age was around 48 years (95% CI: 46.94–49.63). Approximately 6% (95% CI: 4.37-8.14%) of the study population reported having been diagnosed with diabetes while 8.23% (95% CI: 6.39-10.54%) had either a prior diagnosis or had a measured random blood glucose over 11.1 mmol/L during our survey. Over a fifth of the study population reported a prior hypertension diagnosis. When considering both measured and previously diagnosed high blood pressure, 47.69% (95% CI: 44.47-50.91%) were potentially hypertensive. Over one-third of the study population had a measured BMI in the obese range. Only 36.21% (95% CI: 32.85-39.71%) of the study population had neither a reported diagnosis nor a measured NCD of those assessed in the survey. This was the healthy comparison sample for subsequent analyses.

**Table 1:** Population characteristics, adults aged 25 + years (Sample size = 854, estimated population size estimate 11,611)

Variable	Category	Mean or % (95% CI)	Sample size
*Demographics*			
*Age*,* years *(*n = 850)*		48.29 (46.94, 49.63)	850
*Sex*	Male	36.34% (33.18%, 39.63%)	305
	Female	63.66% (60.37%, 66.82%)	549
*Marital status* (*n = 851)*	Currently married/ living together	48.57% (44.22%, 52.94%)	409
	Divorced/separated/ widowed	33.50% (29.47%, 37.79%)	289
	Never married	17.93% (14.74%, 21.64%)	153
*Educational attainment*	No education	10.86% (8.27%, 14.13%)	84
	Basic education	61.42% (57.27%, 65.40%)	535
	Secondary/SHS or tertiary	27.72% (23.37%, 32.55%)	235
*Ethnic group*	Ga-Dangme	77.68% (73.10%, 81.67%)	654
	Akan	12.72% (9.90%, 16.19%)	113
	Other	9.60% (7.49%, 12.23%)	87
*Religion*	No religion	4.99% (3.16%, 7.78%)	43
	Christianity	66.80% (62.10%, 71.19%)	570
	Islam	12.85% (10.22%, 16.03%)	109
	Traditional/spiritual/ other	15.36% (11.50%, 20.22%)	128
*Employment status*	Not working	26.96% (23.24%, 31.03%)	226
	Currently working	73.04% (68.97%, 76.76%)	628
*Household wealth tertile *(*n = 872)*	Most poor	33.00% (27.80%, 38.64%)	281
	Poor	32.26% (27.27%, 37.69%)	290
	Least poor	34.74% (29.64%, 40.22%)	301
*Health indicators*			
Self-reported diagnosis of diabetes	Yes	5.99% (4.37%, 8.14%)	51
	No	94.01% (91.86%, 95.63%)	803
Diabetes (self-reported diagnosis or high measured blood glucose)	Yes	8.23% (6.39%, 10.54%)	72
	No	91.77% (89.46%, 93.61%)	782
Self-reported diagnosis of hypertension	Yes	20.96% (17.57%, 24.80%)	184
	No	79.04% (75.20%, 82.43%)	670
Hypertension (self-reported diagnosis or high measured blood pressure)	Yes	47.69% (44.47%, 50.91%)	404
	No	52.31% (49.09%, 55.53%)	450
Obesity (measured BMI *≥* 30 kg/m^2^) (*n* = 850)	Yes	35.13% (31.31%, 39.15%)	295
	No	64.87% (60.85%, 68.69%)	555
Healthy sample (no NCD) (*n* = 851)	Yes	36.21% (32.85%, 39.71%)	306
	No	63.79% (60.29%, 67.15%)	545
*Psychosocial indicators*			
*Quality of Life*			
Overall perception of quality of life	Very poor	1.19% (0.58%, 2.41%)	8
	Poor	8.51% (6.50%, 11.06%)	69
	Neither poor nor good	35.77% (31.29%, 40.51%)	298
	Good	45.14% (39.81%, 50.58%)	394
	Very good	9.40% (6.73%,12.98%)	85
Overall perception of health	Very dissatisfied	1.12% (0.54%, 2.31%)	9
	Dissatisfied	6.12% (4.35%, 8.54%)	55
	Neither satisfied nor dissatisfied	18.18% (15.32%, 21.45%)	144
	Satisfied	61.46% (56.99%, 65.74%)	528
	Very satisfied	13.12% (10.08%, 16.90%)	118
Physical Health (0-100)		72.15 (70.96, 73.34)	
Psychological (0-100)		70.32 (68.61, 72.04)	
Social relationships (0-100)		66.95 (65.22, 68.69)	
Environment (0-100)		57.37 (55.87, 58.86)	
*Psychological distress score* *(9–45)*		20.33 (19.77, 20.89)	
*Oslo Social Support Scale* *(3–14)*	Poor social support	44.79% (39.68%, 50.01%)	383
	Moderate social support	41.61% (37.56%, 45.78%)	360
	Strong social support	13.60% (10.36%,17.66%)	111
*Average Perceived Stress Scale Score (0–4)*		1.69 (1.64, 1.74)	

In terms of QoL and psychosocial indicators, most of the study population reported having a good or very good overall perception of their QoL and the substantial majority (nearly 75%) reported being either satisfied or very satisfied with their health. Participants scored highest on the physical health domain of QoL (over 72 points on a scale of 0 to 100), followed by psychological and social relationships, with environment being lowest-ranking domain at around 57 points (95% CI: 55.87–58.86). Psychological distress scores averaged around 20 points on a scale from 9 to 45, while perceived stress scores had a mean of approximately 1.7 on a scale of 0 to 4 (or about 17 on the original scale of 0 to 40). Nearly 45% of the study population was classified as having poor social support.

Table 2 shows results of hypothesis tests for significant differences between each of the NCD related groups and the healthy comparison group in QoL and psychosocial wellbeing. In bivariate analyses, differences between the diabetes, hypertension and obesity groups compared to the healthy group were only statistically significant for QoL indicators and not for the psychosocial measures of distress, social support, and perceived stress. Sensitivity analyses looking separately at the distress/lack of control and lack of self-efficacy dimensions of the PSS-10 also did not show any evidence of associations with NCD status.

**Table 2:** Bivariate associations between NCD conditions, quality of life and psychosocial wellbeing

Variable	Healthy comparison group (*n* = 306)	Diabetes (diabetes/ raised blood glucose) (*n* = 72)	Hypertension (diagnosis/ raised blood pressure) (*n* = 404)	BMI *≥* 30 kg/m^2^ (*n* = 295)
	Mean or % (95% CI)	Mean or % (95% CI)	Mean or % (95% CI)	Mean or % (95% CI)
**Quality of Life (QoL)**				
*Overall perception of quality of life*				
Very poor	0.87% (0.20%, 3.73%)	0%	1.48% (0.56%, 3.86%)	1.24% (0.30%, 5.01%)
Poor	9.72% (6.25%, 14.80%)	20.80% (10.19%, 37.78%)	8.36% (5.81%, 11.89%)	5.71% (3.25%, 9.83%)
Neither poor nor good	33.29% (27.13%, 40.08%)	39.98% (26.85%, 54.73%)	37.69% (32.35%, 43.35%)	38.08% (30.61%, 46.15%)
Good	46.80% (39.10%, 54.65%)	30.76% (20.20%, 43.80%)	43.62% (37.35%, 50.10%)	43.92% (37.00%, 51.09%)
Very good	9.33% (5.88%, 14.50%)	8.47% (3.72%, 18.12%)	8.84% (5.83%, 13.18%)	11.05% (7.14%, 16.72%)
*Overall perception of health*		*	*	*
Very dissatisfied	0.43% (0.06%, 3.08%)	**3.07% (0.41%**,** 19.48%)**	**1.08% (0.37%**,** 3.17%)**	**1.28% (0.38%**,** 4.19%)**
Dissatisfied	5.80% (3.55%, 9.35%)	**13.03% (6.72%**,** 23.77%)**	**7.37% (4.54%**,** 11.73%)**	**4.16% (2.17%**,** 7.85%)**
Neither satisfied nor dissatisfied	11.54% (7.93%, 16.51%)	**22.13% (13.41%**,** 34.27%)**	**22.17% (17.99%**,** 27.01%)**	**23.35% (17.51%**,** 30.42%)**
Satisfied	65.69% (58.89%, 71.91%)	**52.16% (40.99%**,** 63.11%)**	**58.00% (52.63%**,** 63.18%)**	**59.48% (53.03%**,** 65.62%)**
Very satisfied	16.52% (11.81%, 22.63%)	**9.61% (4.24%**,** 20.35%)**	**11.38% (7.95%**,** 16.04%)**	**11.73% (7.76%**,** 17.35%)**
*QoL domains*				
* Physical Health (0-100)*	75.50 (73.94, 77.05)	**63.42 (58.48**, 68.36)***	**69.40 (67.58**, 71.23)***	**70.67 (68.94**, 72.40)***
* Psychological (0-100)*	71.77 (69.74, 73.80)	**67.09 (62.83**, 71.35)*	**69.25 (67.15**, 71.36)*	70.69 (68.19, 73.19)
* Social relationships (0-100)*	67.66 (65.57, 69.76)	**63.04 (58.96**, 67.12)*	67.05 (64.89, 69.20)	66.83 (64.00, 69.66)
* Environment (0-100)*	57.65 (55.79, 59.51)	**53.76 (50.54**, 56.98)*	57.24 (55.49, 58.99)	58.26 (56.18, 60.34)
***Psychological distress score (9–45)***	20.06 (19.26, 20.87)	*21.41 (19.91*,* 22.90)*	20.44 (19.66, 21.23)	20.82 (19.90, 21.73)
***Oslo Social Support Scale***				
Poor social support	45.16% (37.68%, 52.86%)	47.93% (35.42%, 60.70%)	43.91% (37.68%, 50.33%)	42.66% (35.31%, 50.35%)
Moderate social support	41.59% (35.47%, 47.98%)	42.85% (31.65%, 54.83%)	42.90% (37.55%, 48.42%)	42.98% (36.18%, 50.05%)
Strong social support	13.25% (8.84%, 19.41%)	9.22% (4.39%, 18.35%)	13.19% (9.59%, 17.89%)	14.36% (9.97%, 20.26%)
***Average Perceived Stress Scale score (0–4)***	1.69 (1.61, 1.78)	1.68 (1.58, 1.79)	1.68 (1.62, 1.73)	1.73 (1.67, 1.80)

Notes: Each disease category is compared to the healthy comparison group in column 2. *Italics indicate p-values less than 0.10*. * indicates*p*-values < 0.05. ** indicates p-values < 0.01. *** indicatesp-values < 0.001

Scores shown in Table 2 suggest that QoL was generally lower among individuals living with obesity, hypertension or diabetes compared to the healthy comparison group. Individuals living with diabetes had significantly reduced scores across all QoL domains compared to the healthy comparison group, whilst significant differences were only observed for physical health and psychological domains among those with hypertension, and only physical health among those with obesity. Supplementary analyses examining those specifically with a diagnosis of diabetes or hypertension show that those reporting a hypertension diagnosis had lower average scores than all those with hypertension (diagnosed or measured), and those reporting a diabetes diagnosis tended to have lower average scores than all those with diabetes (diagnosed or measured) (see Additional file 1).

Given that statistically significant differences were only observed between the healthy and NCD groups on QoL indicators, multivariable analyses were only conducted for the QoL outcomes. Table 3 presents risk ratios and beta coefficients for comparisons of diabetes status, hypertension status and obesity against the healthy population group for each of the six QoL outcomes, while controlling for sociodemographic covariates. (For full model results, see Additional files 2, 3, 4, 5, 6 and 7).

Adults with diabetes (either a self-reported diagnosis or measured random blood glucose over 11.1 mmol/L), with hypertension (self-reported diagnosis or measured high blood pressure) or with a BMI over 30 kg/m^2^ were significantly less likely to be satisfied or very satisfied with their health compared to adults without any NCD condition [Risk Ratios: 0.79 (95% CI: 0.63–0.98), 0.87 (95% CI: 0.79–0.95) and 0.87 (95% CI: 0.77–0.97) for diabetes, hypertension and obesity, respectively]. Moreover, adults with diabetes and obesity had significantly reduced scores in the physical health domain of QoL compared to those without an NCD. Those with diabetes scored on average 8.27 points lower (95% CI: -13.55– -2.99) than the healthy group in the physical health domain of QoL while those with obesity scored 2.74 points lower on average (95% CI: -5.15– -0.32); these differences were statistically significant with *p* < 0.01 for diabetes and *p* < 0.05 for obesity. Adults with hypertension scored on average 2.32 points lower (95% CI: -4.84–0.21) on physical health QoL compared to the healthy group and this difference had borderline significance (*p* < 0.10).

In additional analyses looking specifically at self-reported diagnosis of diabetes and hypertension, those reporting a diagnosis of diabetes or hypertension had more pronounced reductions in the likelihood of being satisfied/very satisfied with their health and in their scores for the physical health domain of QoL compared with the healthy group, as well as a tendency toward reduced scores in other domains of QoL. Other factors that remained associated with QoL outcomes in the multivariable models included age, sex, marital status, education, employment status, wealth tertile and religion (see Additional files 2, 3, 4, 5, 6 and 7).

**Table 3:** Associations between quality-of-life (QoL) outcomes and diabetes, hypertension, and obesity among adults in Ga Mashie aged 25 + years

	Overall QoL perception (Outcome: Good/very good)	Health satisfaction (Outcome: Satisfied/very satisfied)	Domain 1: Physical health	Domain 2: Psychological	Domain 3: Social	Domain 4: Environmental
	Risk ratio (RR) (95% CI)	Risk ratio (RR) (95% CI)	Coefficient (95% CI)	Coefficient (95% CI)	Coefficient (95% CI)	Coefficient (95% CI)
**Model coefficients: Diabetes vs. healthy group (** *n* ** = 374)**	0.86 (0.61, 1.22)	0.79* **(0.63**,** 0.98)**	-8.27** **(-13.55**,** -2.99)**	-2.17 (-6.89, 2.55)	-2.56 (-7.79, 2.67)	-2.69 (-6.64, 1.25)
**Model coefficients: Hypertension vs. healthy group** (*n*** = 702)**	1.01 (0.85, 1.19)	0.87*** **(0.79**,** 0.95)**	*-2.32* *(-4.84*,* 0.21)*	-1.36 (-3.60, 0.88)	0.57 (-2.21, 3.35)	0.25 (-1.82, 2.31)
**Model coefficients: Obesity vs. healthy group** (*n*** = 594)**	1.00 (0.82, 1.23)	0.87* **(0.77**,** 0.97)**	-2.74* **(-5.15**,** -0.32)**	-0.78 (-3.25, 1.69)	-1.04 (-4.23, 2.15)	0.39 (-2.06, 2.84)

Notes: All models control for sex, age, marital status, educational attainment, employment status, household wealth tertile, religious affiliation and ethnic group. Risk ratios for overall QoL perception and health satisfaction were obtained from Poisson regression models. Coefficients for the four domains of QoL were obtained from linear regression models. Italics indicate *p*-values less than 0.10. * indicates *p*-values < 0.05. ** indicates *p*-values < 0.01. *** indicates *p*-values < 0.001

## Discussion

Policy and research interests in health-related QoL have increased recently, and this is precipitated by a growing body of evidence suggesting that psychosocial factors significantly influence health outcomes and self-management health behaviours. Against this backdrop, we sought to examine the relationships between NCDs, QoL and psychosocial wellbeing among a representative sample in the urban community of Ga Mashie, Accra, Ghana. Our results show that most of the study participants report having a good or very good overall perception of their QoL and a substantial majority (nearly 75%) also report being either satisfied or very satisfied with their health. Participants scored highest on the physical health domain of QoL, followed by psychological and social relationships, with the environment being the lowest-ranking domain. No differences in psychosocial wellbeing indicators are observed between any of the diabetes, hypertension or obesity groups and the ‘healthy’ comparison groups. This could mean that these groups truly do not differ in terms of psychological and social wellbeing in this community. Alternatively, there may be methodological factors affecting this result, such as if the instruments do not adequately measure social support, psychological distress or perceived stress in this population or if there are confounding factors or limited statistical power. However, we do see significant differences in QoL indicators. In particular, adults with diabetes, hypertension and obesity are significantly less likely to report being satisfied or very satisfied with their health compared to adults without any reported or measured NCD. Moreover, adults with diabetes and obesity have significantly reduced scores in the physical health domain of QoL compared to those without an NCD. Adults with hypertension have marginally lower scores on physical health QoL compared to the healthy group.

The literature on NCDs continues to expand and now goes beyond the evaluation of clinical outcomes to dimensions such as patient-reported outcome measures such as QoL. The analysis presented here gives evidence of a relationship existing between NCDs such as diabetes, hypertension, and obesity and reduced health-related QoL. Similarly, other research has reported relationships between these NCDs or other physical health conditions and diminished QoL [45–49]. For example, in a study in Spain, Banegas and colleagues reported that older adult patients with diabetes, hypertension and obesity reported reduced health-related QoL compared to those without these conditions [50].

In addition, our supplementary analyses show even greater reductions in health-related and other QoL measures when looking specifically at the subset of the study population reporting a *diagnosis* of diabetes or hypertension (excluding those with *only* measured high blood glucose or measured high blood pressure). Further research in this setting may seek to understand what aspects of having a diagnosis are associated with further reductions in QoL, whether it may be disease severity, treatment regimens, knowledge and awareness of disease or other factors. For example, a study in Bangladesh found that among patients with Type 2 diabetes, the presence of complications such as retinopathy and neuropathy was associated with lower scores in all domains of the WHOQOL-BREF [46], suggesting the potential importance of disease severity in QoL. A study among patients with hypertension in Nigeria found that those taking one to three medications scored higher in terms of health-related QoL than those taking more than three medications [51]. Research in Saudi Arabia [52] also found treatment adherence to be associated with better QoL among patients with diabetes or hypertension. Further study in Ga Mashie may help to identify which particular groups of people with NCDs are at highest risk for reduced QoL and the factors that may be driving these reductions.

In addition to NCD status, other sociodemographic factors were observed to be associated with QoL in our study population. Notably, individuals belonging to higher wealth tertiles and with higher levels of education consistently scored higher on various indicators of QoL. Studies in other countries such as Portugal and Pakistan have likewise reported higher QoL among the population for those with higher socio-economic status [53, 54]. This finding supports social determinants of health frameworks which show socioeconomic gradients in health, with higher socioeconomic position often being associated with better health outcomes [55]. Such socioeconomic gradients in QoL may be due to the material and psychosocial advantages those with higher wealth and education may have, such as access to greater resources or reduced stress.

Driven by urbanization, nutrition and economic transitions, the burden of obesity, hypertension, diabetes and associated NCDs is expected to increase globally, with the largest increases anticipated in the most resource-poor settings in the world including in sub-Saharan Africa [2]. Within this context, our study highlights an urgent need for interlinked transdisciplinary research, health service and policy agendas to describe, understand and mitigate the implications of the NCD burden on population health and wellbeing. This demands careful examination of the social, cultural, economic, and environmental determinants of the NCD burden and inequity of risk exposure in different contexts. This is likely to include further efforts to understand mechanisms of effect between disease and psychosocial wellbeing and QoL to identify and leverage enablers to generally positive perceptions of wellbeing and QoL – as we observed – whilst diminishing barriers to better health. This holistic approach will optimize patient health and QoL outcomes by addressing the interconnected influences of biological, psychological, and social factors in the NCD burden, with significant implications for informing tailored interventions; it will also support programmes to address the emotional and social needs of individuals with NCDs.

### Study limitations

The large sample size, representative sampling design, variety of topics measured, and the inclusion of both self-reported as well as objective measures of health present major strengths of the study. Nevertheless, findings should be interpreted in relation to potential biases in the sample due to non-response, refusals, or inability to locate households. For example, the sample had a preponderance of women. In addition, people without any of the NCDs asked about or measured in the survey (diabetes, hypertension, obesity, heart disease, stroke, chronic lung disease, cancer, asthma, arthritis, kidney disease, liver disease or high blood cholesterol) were classified as ‘healthy’ for the purposes of our analysis, but they may have other conditions affecting their QoL that were not measured in the survey. Finally, the CARE household survey was an observational cross-sectional study which hinders our ability to make any causal inference about the observed associations between QoL and NCDs.

## Conclusions

This study found that individuals with diabetes, hypertension and obesity had significantly reduced measures of health-related QoL outcomes compared to those without an NCD after controlling for sociodemographic characteristics. Specifically, they were less likely to report being satisfied or very satisfied with their health and had lower scores in the physical health domain of QoL. The findings imply that adults with diabetes, hypertension and obesity in Ga Mashie, Accra, are at a higher risk of experiencing reduced health-related QoL. The results highlight the importance of understanding the factors that contribute to reduced QoL among persons with diabetes, hypertension and obesity, and the need to design interventions and coping strategies for improving QoL among individuals with NCDs in this community.

## Electronic supplementary material

Below is the link to the electronic supplementary material.

**Supplementary Material 1**: **Additional file 1**: Description of data: Expanded bivariate analyses of NCDs, QoL and psychosocial wellbeing.

**Supplementary Material 2**: **Additional file 2**: Models for good/very good overall perception of QoL, controlling for demographic factors; Description of data: Full model results for the outcome of good/very good overall perception of quality of life (QoL) for select NCD categories compared to the healthy group (no NCD).

**Supplementary Material 3**: **Additional file 3**: Models for overall satisfaction with health (satisfied/very satisfied), controlling for demographic factors; Description of data: Full model results for the outcome of satisfied/very satisfied with health for select NCD categories compared to the healthy group.

**Supplementary Material 4**: **Additional file 4**: Models for WHO-QOL Domain 1 score: Physical Health, controlling for demographic factors; Description of data: Full model results for the outcome of physical health domain scores of QoL for select NCD categories compared to the healthy group.

**Supplementary Material 5**: **Additional file 5**: Models for WHO-QOL Domain 2 score: Psychological, controlling for demographic factors; Description of data: Full model results for the outcome of psychological domain scores of QoL for select NCD categories compared to the healthy group.

**Supplementary Material 6**: **Additional file 6**: Models for WHO-QOL Domain 3 score: Social, controlling for demographic factors; Description of data: Full model results for the outcome of social domain scores of QoL for select NCD categories compared to the healthy group.

**Supplementary Material 7**: **Additional file 7**: Models for WHO-QOL Domain 4 score: Environmental, controlling for demographic factors; Description of data: Full model results for the outcome of environmental domain scores of QoL for select NCD categories compared to the healthy group.

## Data Availability

Data and materials are available from corresponding author on request.

## Abbreviations

EA: Enumeration area
BMI: Body mass index
NCD: Non-communicable disease
ODK: Open Data Kit
QoL: Quality of life
WHO: World Health Organisation
